# Characterizing Variability of Modular Brain Connectivity with Constrained Principal Component Analysis

**DOI:** 10.1371/journal.pone.0168180

**Published:** 2016-12-21

**Authors:** Jun-ichiro Hirayama, Aapo Hyvärinen, Vesa Kiviniemi, Motoaki Kawanabe, Okito Yamashita

**Affiliations:** 1 Brain Information Communication Research Laboratory Group, Advanced Telecommunications Research Institute International (ATR), Kyoto, Japan; 2 Department of Computer Science/HIIT, University of Helsinki, Helsinki, Finland; 3 Gatsby Computational Neuroscience Unit, University College London, London, United Kingdom; 4 Department of Diagnostic Radiology, Oulu University Hospital, Oulu, Finland; 5 RIKEN Center for Advanced Integrated Intelligence Research, Japan; University of Texas at Austin, UNITED STATES

## Abstract

Characterizing the variability of resting-state functional brain connectivity across subjects and/or over time has recently attracted much attention. Principal component analysis (PCA) serves as a fundamental statistical technique for such analyses. However, performing PCA on high-dimensional connectivity matrices yields complicated “eigenconnectivity” patterns, for which systematic interpretation is a challenging issue. Here, we overcome this issue with a novel constrained PCA method for connectivity matrices by extending the idea of the previously proposed orthogonal connectivity factorization method. Our new method, modular connectivity factorization (MCF), explicitly introduces the modularity of brain networks as a parametric constraint on eigenconnectivity matrices. In particular, MCF analyzes the variability in both intra- and inter-module connectivities, simultaneously finding network modules in a principled, data-driven manner. The parametric constraint provides a compact module-based visualization scheme with which the result can be intuitively interpreted. We develop an optimization algorithm to solve the constrained PCA problem and validate our method in simulation studies and with a resting-state functional connectivity MRI dataset of 986 subjects. The results show that the proposed MCF method successfully reveals the underlying modular eigenconnectivity patterns in more general situations and is a promising alternative to existing methods.

## 1 Introduction

Characterizing the variability of the brain’s functional network organization is of fundamental importance in basic neuroscience as well as clinical researches. Functional connectivity [1] is usually measured as pairwise covariances or correlations of neural signals, typically for 5-10 minutes of resting state, and the connectivities among many brain regions are summarized as what is called the (functional) connectivity matrix. The inter-subject variability of this matrix, or its associated graph, has been linked to individual differences in cognitive function and neurological health or disease (e.g., [2–4]); the variability is observed even within a single subject in a shorter time-scale, either during rest or correspondingly to the changes in task states (e.g., [5–11]), further indicating its relation to ongoing mental states. The central issue is thereby to discover characteristic patterns from connectivity matrices collected from many subjects and/or time windows, for which unsupervised machine learning or exploratory multivariate analysis techniques play a crucial role.

Principal component analysis (PCA) is one such unsupervised method that has been successfully applied to analyzing the variability of functional connectivity matrices, as first demonstrated by [8] with resting-state functional magnetic resonance imaging (fMRI). They showed that principal components (PCs) and the associated basis patterns, called *eigenconnectivity*, shed unique light on the intrinsic structures of variability that complements other approaches (e.g., using *k*-means clustering, as in [7, 9]). In subsequent studies, more advanced methods such as independent component analysis (ICA) [12] have also been used for a similar purpose, while the intuitive notion of eigenconnectivity retains its fundamental importance to characterize the variability of connectivity matrices.

However, in practice, performing PCA-based eigenconnectivity analysis on high-dimensional connectivity data has a severe limitation; the visualization and interpretation of complicated eigenconnectivity patterns are often quite challenging. Each pattern is typically visualized as a matrix using any appropriate color mapping or as a graph superimposed on an anatomical brain image where each node represents a brain region [8]. In either case, the visualized patterns can be very complicated when the number of nodes is large, and often further simplifications or post-hoc analysis of those patterns are needed to draw meaningful conclusions.

Here, we overcome such difficulties with a novel constrained PCA approach that explicitly takes the brain’s functional modularity into account. The notion of functional network modules in the brain has already been widely accepted, as exemplified by resting-state networks (RSNs) [13, 14], and a modular view is often very helpful to intuitively understand complicated relationships among a large number of network nodes. In particular, our approach naturally allows a systematic visualization using the modules’ spatial patterns which are usually more intuitive than graphical ones; this is a great advantage over standard PCA or more conventional constrained PCA techniques such as sparse PCA (e.g., [15, 16]) and 2D-PCA [17]. Such an idea of analyzing network variability in terms of underlying modules is therefore very attractive from both neurophysiological and practical perspectives.

Previously, we developed one such module-constrained PCA technique called *orthogonal connectivity factorization (OCF)* [18]. OCF decomposes the variability of the connectivity matrices into those of pairwise *inter-module* connectivities, i.e., connectivity between two groups of nodes. The two groups (herein called *modules*) are found in a data-driven manner by learning two corresponding weight vectors that are mutually orthogonal, each of which roughly defines the spatial extent of a module. In our application to resting-state fMRI connectivity data, optimized weight vectors produced meaningful spatial patterns resembling well-known RSNs, giving a compact and interpretable decomposition of connectivity matrices. However, OCF aims at a very simple analysis and visualization of the variability of connectivity, which leads to some lack of generality.

In the present study, we extend the OCF approach one step further and develop a new module-constrained PCA method called *modular connectivity factorization (MCF)*. Like OCF, this new method finds network modules to decompose the variability of connectivity matrices, while it improves OCF in a number of ways. Most importantly, MCF analyzes the variabilities of both *inter-module* connectivity and *intra-module* connectivity, i.e., connectivity within each single module. In fact, OCF implicitly assumes that the variability of intra-module connectivity is negligible, which may cause misinterpretation of results when such an assumption does not hold. To further improve the interpretability, we also constrain the weight vectors in MCF so that the same signs are in each module. Moreover, we generalize MCF so that it can learn eigenconnectivities among more than two modules to give added flexibility for representing eigenconnectivity patterns.

Our method is based on an explicit factorization of eigenconnectivity matrices with module weight vectors and a matrix summarizing the eigenconnectivity at the module level. The weight vectors are constrained so that the modules are mutually non-overlapped, which is a stronger assumption than orthogonality in OCF. Further nonnegativity constraints on the weight vectors avoid mixed signs in each module, and the factorized representation can be readily extended with more than two modules. We solve the optimization problem of our constrained PCA using an approximate gradient projection technique, in conjunction with an efficient initialization scheme based on post-hoc analysis of the eigenconnectivity patterns obtained by PCA.

The main goals of this study are summarized as follows. We 1) present the theory and algorithms of MCF with sufficient mathematical details and 2) show MCF’s advantages over existing eigenconnectivity analysis methods, namely PCA and OCF. For the second purpose, we specifically perform simulation studies by artificially creating connectivity matrices so that the ground truth is known. To further demonstrate the method’s applicability to real data, we also 3) apply our method to a publicly available resting-state fMRI connectivity dataset and compare the result with other methods.

## 2 Materials and methods

In this section, we first introduce the two existing methods for eigenconnectivity analysis (PCA and OCF), and then describe the underlying motivations and mathematical details of our new MCF method. Subsequently, we explain the details of our simulation studies and real-data analysis which we performed to compare these methods. Table 1 summarizes some common notations used below.

**Table 1:** List of notations.

Symbol	Description
***X***_*n*_	*D* × *D* connectivity matrix (*n* = 1, 2, …, *N*)
$\bar{X}$	Sample average of connectivity matrices
${\tilde{X}}_{n}$	Centered connectivity matrix $X_{n}-\bar{X}$
***B***	Eigenconnectivity matrix
*s*_*n*_	Corresponding component of *n*-th connectivity matrix
***W***	*D* × *K* module weight matrix
***w***_*k*_	Weight vector of *k*-th module; *k*-th column of ***W***
***G***	*K* × *K* module-level eigenconnectivity matrix
*g*_*kl*_	Eigenconnectivity between modules *k* and *l*; (*k*, *l*)-entry of ***G***
Ω	Set of ***W***s that have at most one nonzero element per row; see Eq (6)
Ω_+_	Set of ***W***s that are nonnegative and in Ω; see Eq (8)
‖ ⋅ ‖	*ℓ*_2_-norm (for vector) or Frobenius norm (for matrix)
tr[⋅]	Trace of a matrix
^⊤^	Transpose of a matrix
R	Set of real numbers
$R_{+}$	Set of nonnegative real numbers

### 2.1 Principal component analysis (PCA)

We begin by introducing the basic concept of eigenconnectivity analysis using PCA.

Denote by $X=\left(x_{ij}\right)\in R^{D\times D}$ a connectivity matrix of interest representing the pairwise connectivity among a predefined set of *D* nodes (e.g., brain regions). Each element *x*_*ij*_ denotes the connectivity between nodes *i* and *j* quantified with any appropriate measure. Diagonal elements *x*_*ii*_ (i.e., self-connections) are also allowed to vary for generality. In the present study, we focus on a symmetric (undirected) connectivity measure, where ***X*** is a symmetric matrix. Since we observe many connectivity matrices, we can in fact consider ***X*** a random quantity (random matrix) from which we observe a sample.

Our goal here is to decompose the variability of the observed connectivity matrices into interpretable components for which PCA serves as the most fundamental method. Specifically, given sample {***X***_1_, ***X***_2_, …, ***X***_*N*_} that consists of *N* instances of random matrix ***X***, PCA finds a linear decomposition, such that

$$\begin{array}{c} X_{n}=\bar{X}+s_{1n}B_{1}+s_{2n}B_{2}+\cdots , \end{array}$$ (1)

where $\bar{X}$ denotes the sample mean, *s*_*mn*_ denotes the *m*-th principal component (PC) for *n* = 1, 2, …, *N*, and ***B***_*m*_ denotes the corresponding pattern of the loadings, i.e., an eigenconnectivity matrix. Patterns ***B***_1_, ***B***_2_, … can be extracted one-by-one using a well-known deflation method, so that the *m*-th component maximally explains the residual sample variance that was not explained by the preceding components.

We focus on the first deflation step, in which we solve the following maximization problem to obtain pattern ***B*** (where the subscript is omitted for simplicity):

$$\begin{array}{c} \max_{B}\sum_{n}{\left(\text{tr}[B^{⊤}{\tilde{X}}_{n}]\right)}^{2},\;\text{subject}\;\text{to}\;∥B∥=1, \end{array}$$ (2)

where ${\tilde{X}}_{n}:=X_{n}-\bar{X}$ is the centered connectivity matrix and $∥B∥:=\sqrt{\sum_{ij}b_{ij}^{2}}$ denotes the Frobenius norm. Here, $\text{tr}[B^{⊤}\tilde{X}]$ is the dot-product between matrices ***B*** and $\tilde{X}$. Thus the objective function simply means (*N* times) the sample variance along the one-dimensional subspace spanned by ***B***. Note that if every ***X***_*n*_ is symmetric, optimal ***B*** is always symmetric, as is easily verified; we omit the explicit symmetricity constraint in Eq (2) for simplicity. In the subsequent deflation steps, the same problem is recursively solved, where the component, explained in the preceding step, is further subtracted from every ${\tilde{X}}_{n}$.

### 2.2 Orthogonal connectivity factorization (OCF)

The above idea of PCA-based eigenconnectivity analysis [8] is fundamental to characterize the variability of brain connectivity. However, since the obtained high-dimensional eigenconnectivity patterns ***B*** can be very complicated, their visualization and interpretation are quite challenging.

For example, as in [8] and in some examples below in the present study, we typically visualize each matrix pattern as a tiled square colored according to the elements’ values. However, such a simple visualization might not be very intuitive without a succinct one-dimensional ordering of nodes; it may even be problematic due to the arbitrariness of the ordering. The pattern can also be visualized more intuitively as an undirected graph by superimposing it on an anatomical brain image, although the graph drawn can again be very complicated, and ad-hoc pruning of the nodes and/or edges is usually required for simplification.

To overcome such difficulties in PCA eigenconnectivity analyses, OCF [18] introduces a constrained eigenconnectivity matrix ***B*** so that the eigenconnectivity pattern is effectively simplified and naturally admits intuitive interpretations. OCF assumes that each (symmetric) eigenconnectivity matrix ***B*** is at most rank two, according to an empirical observation that the eigenspectrums of ***B*** (obtained by PCA) are often dominated by two eigenvalues with opposite signs [18], and then explicitly parameterizes ***B*** as

$$\begin{array}{c} B=\frac{1}{\sqrt{2}}\left(w_{1}w_{2}^{⊤}+w_{2}w_{1}^{⊤}\right), \end{array}$$ (3)

where ***w***_1_ and ***w***_2_ are weight vectors that are assumed to be orthonormal. The coefficient, $1/\sqrt{2}$, simply ensures ‖***B***‖ = 1. Substituting Eq (3) into the original PCA problem Eq (2) (and with the symmetricity of ${\tilde{X}}_{n}$), we have the optimization problem of OCF given by

$$\begin{array}{cc} \max_{w_{1},w_{2}} & \sum_{n}{\left(w_{1}^{⊤}{\tilde{X}}_{n}w_{2}\right)}^{2},\\ \text{subject}\;\text{to} & ∥w_{1}∥=∥w_{2}∥=1,\;w_{1}^{⊤}w_{2}=0. \end{array}$$ (4)

This problem was solved in [18] using a simple alternating maximization algorithm with auxiliary variables. An efficient procedure for an approximate solution has also been developed based on a post-hoc analysis of eigenconnectivity matrices obtained by PCA.

**Fig 1:**
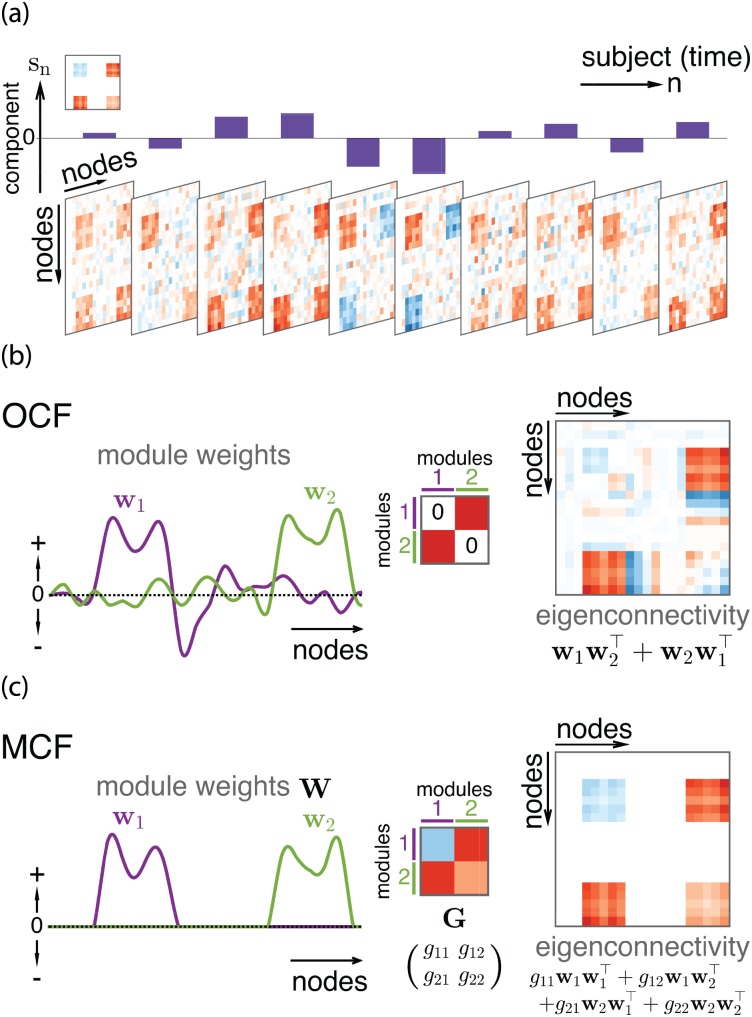
Illustration of OCF and MCF. (a) Single component *s*_*n*_ characterizes variability of connectivity *x*_*ij*_ with a block-structured eigenconnectivity matrix (top left). Here we illustrate a general situation such that diagonal elements *x*_*ii*_ may also vary. (b) OCF’s parametric eigenconnectivity matrix. Two orthogonal weight vectors define two modules, whose symmetrized outer product $w_{1}w_{2}^{⊤}+w_{2}w_{1}^{⊤}$ gives an eigenconnectivity matrix (red: positive, blue: negative). Intra-module eigenconnectivity is assumed to be zero (as illustrated in the middle), but spurious patterns may appear (diagonal parts of eigenconnectivity matrix). (c) Similar illustration for MCF, which explicitly imposes disjointness and nonnegativity on module weights ***W*** = (***w***_1_, ***w***_2_). 2 × 2 real matrix ***G*** fully represents both intra- and inter-module eigenconnectivities; ***WGW***^⊤^ (i.e., the above four terms) gives a node-level eigenconnectivity matrix.

The idea of the specific parameterization of eigenconnectivity matrix Eq (3) is illustrated in Fig 1(a) and 1(b). Here, the observed connectivity matrices are supposed to reflect the modularity of the underlying system, so that each matrix more or less exhibits an approximate block structure under any appropriate node ordering. Then a principal component characterizes the variability in such block-like connectivity matrices (Fig 1(a)). The two weight vectors (Fig 1(b)) have the same length as the number of nodes and in effect define the two modules. The nodes with large absolute weights in ***w***_1_ or ***w***_2_ belong to modules 1 or 2, where the imposed orthogonality is expected to force the modules to be approximately non-overlapping. Then, taking the symmetrized outer products of the two vectors, resultant eigenconnectivity matrix ***B*** mainly explains the variability in an off-diagonal block (and its transpose) of the observed connectivity matrices, which corresponds to the inter-module connectivity between the two modules defined by ***w***_1_ and ***w***_2_.

Hence, OCF specifically seeks a decomposition of the variability into a simple form of eigenconnectivity between any pair of modules. The simplicity of each component pattern is expected to improve the interpretability. Importantly, the weight values of the nodes can be directly mapped to the corresponding spatial positions in the brain so that they can be readily visualized as spatial activation maps on the brain rather than naive matrix representations or complicated graphical patterns. Such a visualization using spatial maps is often more intuitive and does not suffer from arbitrariness in node ordering or methods for graph simplification.

### 2.3 New method: modular connectivity factorization (MCF)

Now we explain our motivation for the new development and describe the formulation and specific algorithms of our MCF method.

#### 2.3.1 Motivation for new method

Although OCF has been demonstrated to be successful in decomposing the variability of connectivity matrices [18], we noticed some of its drawbacks that might prevent it from being widely applicable. The following issues particularly motivated us to develop the new MCF method which we describe below.

First, OCF implicitly assumes that the variability of connectivity within each module (i.e., intra-module connectivity) is almost negligible, where the two self-product terms, $w_{1}w_{1}^{⊤}$ and $w_{2}w_{2}^{⊤}$, are dropped in Eq (3). If this assumption does not hold, the two weight vectors necessarily overlap to spuriously produce nonzero patterns within individual modules (i.e., diagonal blocks of eigenconnectivity matrix; see Fig 1(b)). This inseparability of intra- and inter-module connectivities is problematic because it complicates the interpretation of weight vectors and might even distort the inter-module (off-diagonal) parts of the eigenconnectivity if the intra-module parts need to have large values.

Second, the weight and eigenconnectivity patterns obtained by OCF might contain both positive and negative signs in one module (Fig 1(b)), which is often difficult to interpret. The different signs in eigenconnectivity mean that the connectivities corresponding to positive and negative signs vary in opposite directions. If one increases, the other decreases, and vice versa. Hence, mixed eigenconnectivity signs imply that a module may be divided into submodules with distinct functional properties, characterized by strong anti-correlations (i.e., negative correlations) in the variability of their connectivities to any other modules as well as the variability in their internal connectivities, if intra-module connectivity is also taken into account. Such inhomogeneity would be more interpretable if it were represented explicitly as different modules.

Finally, OCF focuses on the variability of connectivity between a pair of modules, and thus it is not applicable to finding general eigenconnectivity patterns among more than two modules. Although an empirical observation [18] strongly motivated the rank-two approximation, it does not necessarily imply that the information discarded by the approximation is meaningless. In particular, we can expect to obtain eigenconnectivity patterns that are more interesting from any neurophysiological (or any other domain-specific) perspective by increasing the rank and thus the number of modules.

#### 2.3.2 MCF’s representation of eigenconnectivity matrix

We introduce a new parametric representation of simplified eigenconnectivity matrix ***B*** by extending the one of OCF Eq (3). To begin with, we only consider a pair of modules. The general case of more than two modules is described in Section 2.3.5.

The new parametric form of ***B*** is given as follows, which is also illustrated in Fig 1(c):

$$\begin{array}{c} B=\sum_{k=1}^{2}\sum_{l=1}^{2}g_{kl}w_{k}w_{l}^{⊤}=WGW^{⊤}, \end{array}$$ (5)

where ***w***_*k*_ is the weight vector for module *k*, such that node *j* belongs to module *k* when its *j*-th element *w*_*jk*_ is nonzero. The two weight vectors are collected in the *D* × 2 matrix ***W*** = (***w***_1_, ***w***_2_). Real coefficients *g*_*kl*_ determine the relative magnitudes and the signs of these outer products $w_{k}w_{l}^{⊤}$ and are collected in a 2 × 2 matrix ***G*** = (*g*_*kl*_). For simplicity, we assume that matrix ***G*** is non-singular, so that the number of modules equals the rank of ***B***. To avoid an obvious ambiguity in the scaling between ***W*** and ***G***, we fix every ***w***_*k*_ to have unit *ℓ*_2_-norm, i.e., ‖***w***_*k*_‖ = 1 for every *k*.

Next we explain additional assumptions on parameters ***W*** and ***G*** in detail:

1. *Variabilities in both intra- and inter-module connectivities:* As a key difference from the parametrization Eq (3) of OCF, we let every diagonal element *g*_*kk*_ of ***G*** be generally nonzero, so that the new one Eq (5) represents both the *intra-module* and *inter-module* connectivities with outer products $w_{k}w_{k}^{⊤}$ and $w_{k}w_{l}^{⊤}$ (*k* ≠ *l*). Coefficient *g*_*kl*_ specifies the overall strengths and signs of the corresponding term in Eq (5), since the outer products are normalized commonly as ${∥w_{k}w_{l}^{⊤}∥}^{2}=1$. In what follows, we refer to ***G*** as a *module-level eigenconnectivity matrix*, representing node-level ***B*** in a compact form.
2. *Disjointness of modules:* To avoid the spurious effects on intra-module eigenconnectivity due to the overlap of weight vectors among modules, we further constrain the weight vectors so that their nonzero elements do not overlap among modules. That is, we set ***W*** ∈ Ω, where
  $$\begin{array}{cc} \Omega :=\{W\in R^{D\times 2}| & \text{at}\;\text{most}\;\text{one}\;\text{element}\;\text{per}\;\text{row}\;\text{is}\;\text{nonzero}\}. \end{array}$$ (6)
  Then the modules are mutually disjoint, and each $w_{k}w_{k}^{⊤}$ and $w_{k}w_{l}^{⊤}$ (*k* ≠ *l*) represent clearly the intra- and inter-module eigenconnectivity patterns. Note that the weight vectors are now mutually orthonormal (i.e., ***W***^⊤^ ***W*** = ***I***) as in OCF, which readily results from ***W*** ∈ Ω and the unit-norm constraints on the columns as introduced above.
3. *Nonnegativity/Uniform signs:* To improve the interpretability of the obtained weight vectors, we further constrain each module’s weights to have uniform signs. Notice that the sign of each module is actually arbitrary and is eventually absorbed into ***G***. Here, we thus specifically set every weight to be nonnegative, i.e.,
  $$\begin{array}{c} W\in R_{+}^{D\times 2}, \end{array}$$ (7)
  where $R_{+}$ denotes the set of nonnegative real numbers.

The matrix factorization Eq (5) with these specific assumptions as above still has some indeterminacy in parameters ***W*** and ***G***. An obvious indeterminacy is the permutation of the modules. That is, relation ***B*** = ***WGW***^⊤^ holds even if ***W*** and ***G*** are replaced with ***WP*** and ***P***^⊤^ ***GP*** for any permutation matrix ***P*** without violating the above constraints on ***W***. Another indeterminacy is that the global sign of ***G*** can always be flipped since the global sign of ***B*** is immaterial, as in PCA. In practice, these indeterminacies do not seem to cause serious problems, since the permutation and the global sign may be arbitrarily chosen after learning so that the interpretation is easier.

#### 2.3.3 Stepwise MCF via post-hoc matrix factorization

Before presenting the principled constrained PCA approach based on the new eigenconnectivity representation, we first introduce a stepwise approach (Stepwise MCF) to approximately obtain ***W*** and ***G*** based on the eigenconnectivity matrix ***B***_PCA_ precomputed by PCA. The stepwise approach is useful in its own right, while we specifically use it to effectively initialize the iterative algorithm of constrained PCA (see Section 2.3.4). In the following, we briefly sketch the algorithm; the details are found in S1 Appendix (Section A).

Algorithm 1 summarizes the entire procedure of Stepwise MCF to obtain a single MCF component, which essentially consists of the following steps: first, 1) we solve the unconstrained PCA problem Eq (2) to obtain eigenconnectivity matrix ***B***_PCA_, and then 2) compute the two eigenvectors of ***B***_PCA_ corresponding to the largest eigenvalues (in absolute value) to form a *D* × 2 matrix ***U***. Finally, we 3) alternatingly optimize an orthogonal matrix ***V*** and ***W*** ∈ Ω_+_ so that the error between ***UV***^⊤^ and ***W*** is minimized, where

$$\begin{array}{c} \Omega_{+}:=\Omega \cap R_{+}^{D\times 2}. \end{array}$$ (8)

This can be solved by a simple modification of a technique previously developed for (multiclass) spectral clustering [19], and an additional column-wise normalization further ensures ***W*** to satisfy all of the original constraints, i.e., ***W*** ∈ Ω_+_ and ∀*k*‖***w***_*k*_‖ = 1. We further 4) set ***G*** = ***W***^⊤^ ***B***_PCA_ ***W*** to obtain an approximate factorization ***B***_PCA_ ≈ ***WGW***^⊤^.

In this procedure, we randomly initialize ***V*** as an orthogonal matrix and flip the signs of its columns to make every entry of ${\left(UV^{⊤}\right)}^{⊤}1=V\bar{u}$ nonnegative, where $\bar{u}:=U^{⊤}1$ and **1** denotes an all-one vector. This simple heuristics ensures that the columns of ***UV***^⊤^ to be summed up are nonnegative, so that ***UV***^⊤^ becomes closer to nonnegative ***W***. The projection to ***W*** ∈ Ω_+_ possibly results in zero column vectors in ***W*** due to the lack of explicit norm constraint. To avoid this, we detect zero columns to restart the procedure from re-initialized ***V***.

**Algorithm 1** Stepwise MCF (single component)

**Require:** Connectivity matrices {***X***_*n*_}; constant *ϵ* > 0

1: Perform PCA on connectivity matrices to obtain ***B***_PCA_

2: Compute the best rank-two eigenvalue decomposition ***B***_PCA_ ≈ ***UQU***^⊤^; $\bar{u}\leftarrow U^{⊤}1$

3: Randomly generate orthogonal matrix ***V***

4: $V\leftarrow V\text{diag}(\text{sign}\left(V\bar{u}\right))$

5: **repeat**

6:  ***V***_old_ ← ***V***

7:  $W\leftarrow P_{\Omega_{+}}\left(UV^{⊤}\right)$     ▹ Projection to the set Ω_+_ (see S1 Appendix)

8:  **if** any column in ***W*** is zero vector, **then**

9:   Randomly generate orthogonal matrix ***V***

10:   $V\leftarrow V\text{diag}(\text{sign}\left(V\bar{u}\right))$

11:  **else**

12:   Compute singular value decomposition: ***U***^⊤^ ***W*** = ***L*** **Σ** ***R***^⊤^; ***V*** ← ***RL***^⊤^

13:  **end if**

14: **until** $∥V_{\text{old}}^{⊤}V-I∥<\epsilon$

15: Normalize every column in ***W*** to have unit *ℓ*_2_-norm

16: ***G*** ← ***W***^⊤^ ***B***_PCA_ ***W***

We remark that the approximate factorization ***B***_PCA_ ≈ ***WGW***^⊤^ is in fact applicable to any matrix ***B*** other than that obtained by PCA. Thus, Algorithm 1 (except for the first line) can readily be used, e.g., to analyze a single connectivity matrix ***X***_*n*_ or the average $\bar{X}$; the stepwise analysis can even be combined with any methods other than PCA, such as *k*-means clustering (see S2 Appendix) and ICA.

#### 2.3.4 Constrained PCA algorithm

Plugging the factorized eigenconnectivity matrix Eq (5) into the original PCA problem Eq (2), we have the following constrained PCA optimization problem to obtain a single MCF component:

$$\begin{array}{cc} \max_{W,G} & \sum_{n=1}^{N}{\left(\text{tr}[WG^{⊤}W^{⊤}{\tilde{X}}_{n}]\right)}^{2},\\ \text{subject}\;\text{to} & W\in \Omega_{+},\;\forall k\;∥w_{k}∥=1,\;∥G∥=1. \end{array}$$ (9)

The additional unit-norm constraint on ***G*** ensures ‖***B***‖ = 1, since ***W***^⊤^ ***W*** = ***I*** implies ‖***WGW***^⊤^‖ = ‖***G***‖. Note that optimal ***G*** is symmetric because every ${\tilde{X}}_{n}$ is symmetric; if we further constrain ***G*** so that every diagonal entry is zero, constant $1/\sqrt{2}$ in OCF Eq (3) emerges in the off-diagonal entries under the unit-norm constraint on ***G***. An alternative interpretation of Problem (9) is given in S1 Appendix (Section B).

To solve Eq (9), we use a simple alternating maximization scheme with auxiliary variables, similar to [18]. Let ***r*** = (*r*_1_, *r*_2_, …, *r*_*N*_)^⊤^ be an auxiliary vector variable satisfying ‖***r***‖ = 1, and define $C:=\sum_{n}r_{n}{\tilde{X}}_{n}$. Then the square root of the objective function in Eq (9) has the following lower bound:

$$\begin{array}{c} {\left(\text{Objective}\;\text{in}\;(9)\right)}^{\frac{1}{2}}\geq \sum_{n=1}^{N}r_{n}\text{tr}\left[WG^{⊤}W^{⊤}{\tilde{X}}_{n}\right]=\text{tr}\left[WG^{⊤}W^{⊤}C\right], \end{array}$$ (10)

where equality holds when *r*_*n*_ equals $\text{tr}[WG^{⊤}W^{⊤}{\tilde{X}}_{n}]$ times a constant factor to ensure ‖***r***‖ = 1. Since taking the square root does not change the solution of the Problem (9), the Problem (9) is equivalent to the maximization of the right-hand side in Eq (10). The lower bound is then maximized alternatingly between (***W***, ***G***) and ***r***.

The solution for ***r*** was given above. To derive the solution for (***W***, ***G***), we first analytically solve for ***G***, yielding ***G*** = ***W***^⊤^ ***CW***/‖***W***^⊤^ ***CW***‖, and then substitute it into the right-hand side of Eq (10). We again square the objective without changing the optimal solution and eventually have a reduced problem only for ***W***:

$$\begin{array}{c} \max_{W}{∥W^{⊤}CW∥}^{2},\;\text{subject}\;\text{to}\;W\in \Omega_{+},\;\;\forall k\;∥w_{k}∥=1, \end{array}$$ (11)

which we solve with a gradient projection technique (see, e.g., [20]). Note that we do not need to perfectly solve this inner maximization Eq (11), but we only need to increase the objective. In practice, we perform the gradient projection step only once at each alternating maximization step.

We introduce a single gradient projection step as the following update of ***W***:

$$\begin{array}{c} W\leftarrow P(W+\eta \Delta W), \end{array}$$ (12)

where matrix Δ***W*** defines the search direction in the space of ***W***, *η* ≥ 0 is a stepsize parameter, and P is the projection operator to keep ***W*** in the feasible set (see below). Denote by *f*(***W***) the objective function in Eq (11) and by ∇_***W***_ *f*(***W***) its gradient, derived in S1 Appendix (Section C) as

$$\begin{array}{c} \nabla_{W}f\left(W\right)=4CWW^{⊤}CW. \end{array}$$ (13)

Then, according to a theory of optimization on manifold [21], we set the search direction as

$$\begin{array}{c} \Delta W=\nabla_{W}f\left(W\right)-W{\left(\nabla_{W}f\left(W\right)\right)}^{⊤}W, \end{array}$$ (14)

which restricts the gradient in the tangent space of the Stiefel manifold (i.e., the set of ***W***s such that ***W***^⊤^ ***W*** = ***I***) at ***W***. We choose the stepsize *η* at each update by a backtracking line search to satisfy Armijo’s condition (e.g., [20]).

The projection operator in Eq (12) still needs to be specified. Unfortunately, exact orthogonal projection P of ***W*** to feasible set {***W***∣***W*** ∈ Ω_+_, ∀*k*‖***w***_*k*_‖ = 1} has no simple solution. Thus we use an approximate method and first project ***W*** to Ω_+_ without norm constraints, followed by the normalization of every column so that each has a unit norm. Note that the first step can be solved by Proposition 1, given in S1 Appendix (Section A), and the subsequent normalization does not violate ***W*** ∈ Ω_+_, so that ***W*** eventually belongs to the feasible set. Algorithm 2 summarizes the overall algorithm.

**Algorithm 2** MCF (single component): solution to constrained PCA Eq (9)

**Require:** *N* connectivity matrices ${\tilde{X}}_{n}$; initial stepsize *η* > 0; *α* ∈ (0, 1) for Armijo condition, *β* ∈ (0, 1) for backtracking line search, and *ϵ* > 0 for stopping the criterion

1: Initialize ***W*** and ***G*** using Algorithm 1

2: **repeat**

3:  $r_{n}\leftarrow \text{tr}\left[W^{⊤}{\tilde{X}}_{n}WG\right]$ for every *n*

4:  Normalize ***r*** to have unit *ℓ*_2_-norm

5:  $C\leftarrow \sum_{n}r_{n}{\tilde{X}}_{n}$

6:  ∇*f*(***W***) ← 4***CWW***^⊤^ ***CW***

7:  Δ***W*** ← ∇*f*(***W***) − ***W***(∇*f*(***W***))^⊤^ ***W***

8:  ***W***_old_ ← ***W***

9:  **repeat**

10:   $W^{\prime }\leftarrow P_{\Omega_{+}}\left(W+\eta \Delta W\right)$     ▹ Projection to the set Ω_+_ (see S1 Appendix)

11:   Normalize each column of ***W***′ to have unit *ℓ*_2_-norm

12:   *η* ← *βη*

13:  **until** *f*(***W***′) ≤ *f*(***W***) + *α*tr[(∇*f*(***W***))^⊤^(***W***′ − ***W***)]

14:  ***W*** ← ***W***′

15: **until** ‖***W***^⊤^ ***W***_old_ − ***I***‖ < *ϵ*

16: Compute ***G*** = ***W***^⊤^ ***CW*** and normalize ***G*** to have unit Frobenius norm

#### 2.3.5 Generalization to *K* modules

So far we have only considered two modules to represent a single eigenconnectivity matrix, ***B***. In fact, the current formulation allows the number of modules to be any positive integer smaller than *D*, which further generalizes MCF. Let *K* denote the number of modules. Then weight matrix ***W*** has *K* columns, and module-level eigenconnectivity ***G*** is a *K* × *K* square matrix. The above algorithms remain valid, except that the rank-2 approximation in line 2 of Algorithm 1 must be replaced with rank-*K* approximation.

As an exploratory analysis method, however, we must usually avoid setting the numbers of modules too large. The standard choice of *K* = 2 is thus reasonable since it gives the minimal case that includes both intra- and inter-module connectivities. We can also increase *K* so that the patterns obtained are more meaningful in terms of domain knowledge, although the module-level eigenconnectivity patterns might quickly become too complicated. We usually set *K* to less than about five so that the result can be easily visualized.

To support the selection of reasonable numbers of modules *K*, we can also examine the eigenvalue spectrum of ***B***_PCA_: Let *q*_1_, *q*_2_, …, *q*_*D*_ be the eigenvalues of ***B***_PCA_, sorted in nonincreasing order. Then the power (squared Frobenius norm) of ***B***_PCA_ is orthogonally decomposed into squared eigenvalues $q_{k}^{2}$. Given the spectrum, we can use any conventional heuristics in the PCA literature to determine which eigenvalues’ contributions $q_{k}^{2}$ are meaningful (see, e.g., [22]). In practice, such an approach can be easily combined with Stepwise MCF (Algorithm 1). In the subsequent optimization of the constrained PCA (Algorithm 2), we simply use the same *K* selected at the initialization stage.

#### 2.3.6 Extraction of multiple components

The solution to Eq (9) gives the first MCF component in terms of ***W*** and ***G***. Similar to PCA, a deflation method can be used to obtain subsequent components, solving Eq (9) in a recursive manner. Given corresponding components $s_{n}=\text{tr}\left[{\left(WGW^{⊤}\right)}^{⊤}{\tilde{X}}_{n}\right]$, we replace every instance ${\tilde{X}}_{n}$ with the residual unexplained by the preceding components:

$$\begin{array}{c} {\tilde{X}}_{n}\leftarrow {\tilde{X}}_{n}-s_{n}WGW^{⊤}, \end{array}$$ (15)

and then the next component’s ***W*** and ***G*** can be obtained by solving Eq (9) again with updated ${\tilde{X}}_{n}$.

According to this deflation scheme, the *m*-th step yields an approximate decomposition of centered connectivity matrices ${\tilde{X}}_{n}$ in terms of basis {***B***_1_, ***B***_2_, …, ***B***_*m*_} as well as the corresponding components denoted by *s*_*mn*_. Note that matrices ***B***_1_, ***B***_2_, …, ***B***_*m*_ are not necessarily orthogonal to each other (i.e., dot-products $\text{tr}[B_{i}^{⊤}B_{j}]$ are not necessarily zero), so that the total variance (in the data space) explained by the *m* components cannot be simply computed by summing up the variances of components *s*_*mn*_ as in a standard way.

Here, following the idea of adjusted total variance [15], which was originally developed for sparse PCA, we evaluate the total explained variance by MCF components with a correction to the non-orthogonality. First we orthogonalize the basis using the well-known Gram-Schmidt method to get $\sum_{m^{\prime }=1}^{m}{\tilde{s}}_{m^{\prime }n}{\tilde{B}}_{m^{\prime }}=\sum_{m^{\prime }=1}^{m}s_{m^{\prime }n}B_{m^{\prime }}$ with orthonormal basis $\left\{{\tilde{B}}_{m^{\prime }}\right\}$ and adjusted components ${\tilde{s}}_{m^{\prime }n}$. Then we compute the adjusted total variance explained by the MCF components by summing the adjusted component variances, i.e., $\left(1/N\right)\sum_{m^{\prime }=1}^{m}(\sum_{n=1}^{N}{\tilde{s}}_{m^{\prime }n}^{2})$.

#### 2.3.7 Relation to nonnegative tensor factorization

Interestingly, our MCF developed above turns out to be closely related to nonnegative tensor factorization (see, e.g., [23]) which has recently also found applications in the brain’s functional network analyses [24]; the goal is rather different from eigenconnectivity analysis. In S3 Appendix, we compare our method with a particular type of nonnegative tensor factorization as well and discuss their fundamental differences.

### 2.4 Simulation studies

We performed simulation studies to compare the performance of our MCF method with existing methods for eigenconnectivity analysis.

#### 2.4.1 Simulation I: illustrative example

First, we performed a simple simulation to illustrate how our new MCF method improves the existing methods for eigenconnectivity analysis, namely, PCA and OCF. In particular, we examined the effect of increasing the variability of intra-module connectivity on the estimated eigenconnectivity and the weight matrix patterns.

Here, connectivity matrices ***X***_*n*_ were created simply by multiplying a random number generated from standard Gaussian distribution to a true eigenconnectivity matrix ***B*** and adding symmetric random noise ***E***_*n*_. The number of nodes was set to *D* = 20. True ***B*** was created as ***B*** = ***WGW***^⊤^, where two unit-norm columns ***w***_1_ and ***w***_2_ were given so that only entries *w*_*j*1_ for *j* ∈ {4, 5, …, 8} and *w*_*j*2_ for *j* ∈ {12, 13, …, 18} were nonzero. These nonzero values were set uniform in each column. Noise ***E***_*n*_ was generated by first sampling the upper triangular part as random Gaussian white noise (SD: 0.3) and copying it into the strictly lower triangular part to form a symmetric matrix. Matrix ***G*** was specifically given as

$$\begin{array}{c} G=(\begin{array}{cc} \sqrt{c/2} & \sqrt{(1-c)/2}\\ \sqrt{(1-c)/2} & \sqrt{c/2} \end{array}), \end{array}$$ (16)

for some values of *c* ∈ [0, 1]. Notice that the sum of the squares in the diagonal and off-diagonal elements satisfy $g_{11}^{2}+g_{22}^{2}=c$ and $g_{12}^{2}+g_{21}^{2}=1-c$. Thus parameter *c* gives the relative strength of the intra-module network variability in the data.

We applied PCA, OCF and MCF (Algorithm 2, *K* = 2) to the collection of connectivity matrices synthesized as above. We used a relatively large sample size, *N* = 10,000, to clarify the difference particularly due to the modeling assumptions in those methods rather than stochastic estimation errors. We implemented all the methods in Matlab; we used *eigs* function for PCA and the original code for OCF (https://www.cs.helsinki.fi/u/ahyvarin/code/ocf/). We set the tolerance parameters *ϵ* reasonably small, i.e., *ϵ* = 10^−12^ in Algorithm 1 and *ϵ* = 10^−6^ in Algorithm 2. In Algorithm 2, we also set the initial stepsize as *η* = 0.01, Armijo parameter *α* = 10^−4^ as suggested in [25], and *β* = 0.5 to halven the stepsize in each backtracking step. These settings were common for all the experiments below.

#### 2.4.2 Simulation II: quantitative comparison

Next, we performed a more quantitative evaluation of the estimation errors by the above methods. Here, we randomly synthesized *N* symmetric matrices ***X***_1_, ***X***_2_, …, ***X***_*N*_ of size 100 × 100 (*D* = 100) according to the following generative model,

$$\begin{array}{c} X_{n}=s_{1n}B_{1}+s_{2n}B_{2}+E_{n}, \end{array}$$ (17)

so that unit-norm symmetric matrices ***B***_1_ and ***B***_2_ define a two-dimensional subspace in the matrix space, along which observed matrices ***X***_*n*_ largely varied. The coefficients, *s*_1*n*_ and *s*_2*n*_, were randomly generated by zero-mean Gaussian distributions with the standard deviations (SDs) given by 1 and 0.6. Random noise ***E***_*n*_ was again generated from Gaussian white noise (SD: 0.3), symmetrized appropriately as above.

Matrices ***B***_1_ and ***B***_2_ were created randomly in each run of the simulation in the following manner. First, we generated a 100 × 10 weight matrix ***W*** by randomly partitioning the *D* nodes into ten non-singleton subsets (this step simply produced modules of moderate sizes; only the four columns in ***W*** were selected and the remaining six were discarded). and sampling the corresponding nonzero weights *w*_*jk*_ from uniform distribution over [0.5, 1.5], followed by renormalizing each column of ***W*** to have unit *ℓ*_2_-norm. Then we randomly chose two columns in ***W*** to form ***W***_1_ and another two columns from the remaining eight columns of ***W*** to form ***W***_2_. Finally, we generated 2 × 2 symmetric matrices ***G***_1_ and ***G***_2_ from the Gaussian distribution, renormalized them to have a unit Frobenius norm, and set $B_{m}=W_{m}G_{m}W_{m}^{⊤}$ for *m* = 1, 2. To generate each ***G***, we either 1) explicitly set the diagonal elements at zero (i.e., *g*_*kk*_ = 0 for any *k*, as OCF assumes) before normalization or 2) randomly sampled those diagonal elements as well.

We compared 1) the standard PCA on connectivity matrices, 2) OCF, 3) Stepwise MCF (Algorithm 1), which was also used to initialize MCF, and 4) MCF, using the projected gradient method (Algorithm 2). We ran these four algorithms in the two different conditions on true ***G*** explained above.

By construction, ***B***_1_ and ***B***_2_ synthesized above were mutually orthogonal and thus serve as the orthonormal basis of the two-dimensional principal subspace. ***B***_1_ and ***B***_2_ gave the true eigenconnectivity patterns of the first and second PCs to each of which corresponding estimate $\hat{B}$ should approach. Thus, as a basic performance measure, we evaluated estimation error $∥B_{m}-{\hat{B}}_{m}∥$ for *m* = 1, 2, i.e., the root mean squared error (RMSE) between the corresponding elements of the two matrices. We ran the simulation 100 times for given sample size *N* and evaluated the estimation error in each of the 100 runs.

### 2.5 Resting-state fcMRI data analysis

We further compared MCF and OCF using a publicly available functional connectivity MRI (fcMRI) dataset.

#### 2.5.1 Dataset

The dataset consisted of a total of 986 functional connectivity matrices, which we obtained from the USC Multimodal Connectivity Database (http://umcd.humanconnectomeproject.org) [26]. We used the data tagged “1000_ Functional_ Connectomes” and removed 17 connectivity matrices from the original 1003 because the files seemed corrupt. Each connectivity matrix corresponded to one subject, consisting of pairwise correlations of blood-oxygen-level dependent (BOLD) signals between 177 functional regions-of-interest (ROIs) that were obtained using the spatially constrained spectral clustering method [27].

#### 2.5.2 Data analysis

We applied both OCF and MCF (*K* = 2) to this dataset and extracted two components with each method. Due to the existence of local optima in the objectives, we used 20 different initial conditions and examined the results that achieved the maximum explained variance. In fact, the result was qualitatively not sensitive to initial conditions; MCF runs deterministically once initialized by the output of Algorithm 1 and we empirically found that Algorithm 1 was quite robust to the initial choice of ***V***.

**Fig 2:**
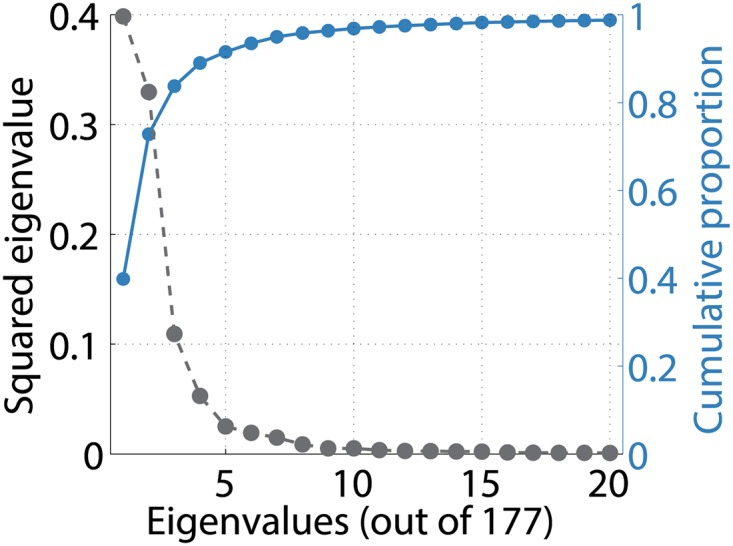
Resting-state fcMRI data. Top 20 squared eigenvalues (black dashed line) of first PCA eigenconnectivity matrix ***B***_PCA_. Their cumulative proportions (with respect to total of 177 eigenvalues) are shown in right vertical axis (blue solid line).

We also demonstrated the generalization of MCF to more than two modules (Section 2.3.5) using the same fcMRI dataset. As a preliminary analysis, we examined the eigenvalue spectrum of the first PCA eigenconnectivity matrix ***B***_PCA_ to roughly estimate a reasonable number of modules *K* (Fig 2). Note that the number of modules in MCF equals the number of eigenvalues retained in the rank-*K* approximation of ***B***_PCA_ ≈ ***UQU***^⊤^ performed in the generalized version of Algorithm 1 (Section 2.3.5). The figure suggested that the first three (84%) or four modules (89%) already cover a large fraction of variability explained by ***B***_PCA_. We thus performed MCF with *K* = 3 and *K* = 4, extracting only one component in each case for simplicity. To avoid local optima, we again used 20 different initial conditions in each *K*, and examined the results that achieved the maximum explained variance.

#### 2.5.3 Visualization

To visualize the spatial weight patterns, we used a simple scheme adopted [18]. We plotted a dot at each spatial coordinate of the ROI in different sizes and colors and displayed the 3D spatial patterns in three different views: from the right lateral, the dorsal, and the posterior. In each view, we projected the 3D dots to the three 2D planes, rather than sectioning the 3D brain, so that the brain is regarded as transparent.

In addition, we illustrated module-level eigenconnectivity ***G*** as an undirected graph with self-loops, where the squared magnitude and the sign of each entry are indicated by the edge width and color. The OCF graph was also drawn in a similar manner, while ***G*** was actually a constant matrix with zero diagonals. Here, we set the global sign of each ***G*** (which is arbitrary) to maximize the sum of the squares of the positive entries. Due to the arbitrariness of the global sign, the signs (and colors) in ***G*** do not indicate excitatory or inhibitory relationships between modules. Rather, they imply that the module-level connectivities of different signs are mutually anti-correlated when varying over individuals.

## 3 Results

### 3.1 Simulation studies

**Fig 3:**
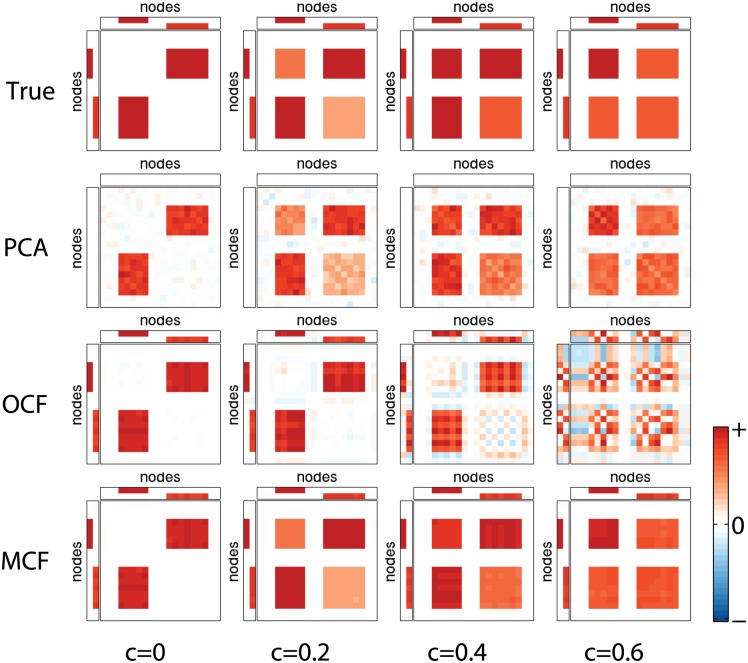
Simulation I: illustrative example. Each panel in the top row shows a true 20 × 2 weight matrix ***W*** = [***w***_1_, ***w***_2_] (as well as its transpose, two small rectangles) and corresponding eigenconnectivity matrix ***B*** (large squares). The four panels differ in relative strength *c* ∈ [0, 1] of intra-module network variability, as indicated at bottom of figure. *c* = 0 implies no variability within each module and greater *c* indicates the stronger intra-module variability; in particular, *c* = 0.6 implies a greater intra-module variability than the inter-module. Bottom three rows show corresponding estimates ***B*** by PCA, OCF, and MCF (proposed method), displayed in the same manner. In all panels, red, white, and blue indicate positive, zero, and negative values, respectively (except for weight matrix rectangles in PCA, which are left blank). Each panel and each ***W*** and ***B*** were scaled individually so that maximum absolute value corresponds to boundary of color range (displayed at bottom right).

As an illustrative example, we first show the true and estimated ***W***s and ***B***s by PCA, OCF and MCF (Simulation I; Fig 3). As seen in the left-most column where only the inter-module connectivity varied (*c* = 0), true pattern ***B*** at the top was successfully recovered by every method. In the second column with a relatively weak variability within modules (*c* = 0.2), PCA and MCF produced almost the same pattern as true ***B***, while the patterns obtained by PCA are relatively noisy compared to those by MCF; as intended, OCF mostly recovered the off-diagonal blocks of true ***B***.

A drawback of OCF is seen when the relative strength of intra-module variability increased. OCF spuriously produced the intra-module part (diagonal blocks) of ***B***, which completely destroyed the other inter-module part (off-diagonal blocks), as seen in *c* = 0.6, caused by the inseparability of the two parts. In comparison, MCF successfully recovered true pattern ***B*** as well as weight matrix ***W*** at every choice of *c*, which clearly illustrates the advantage of separately modeling the intra-module and inter-module network variabilities.

**Fig 4:**
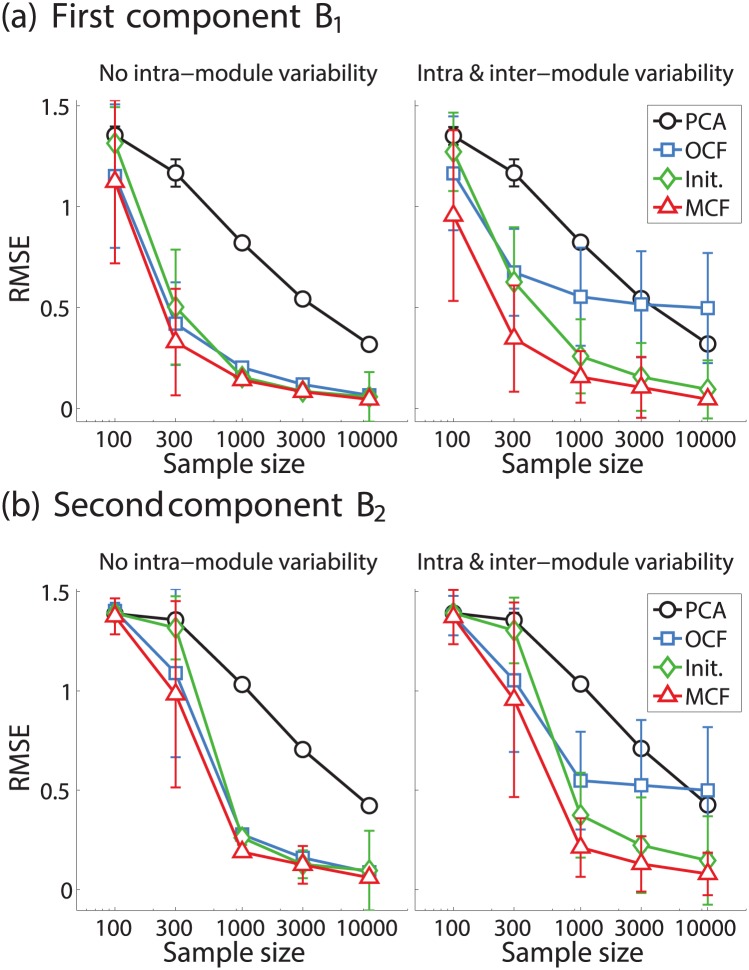
Simulation II: quantitative comparison. (a) Root mean squared error (RMSE) between true and estimated ***B*** of first component versus sample size *N* (in log scale). Left panel: only inter-module connectivity between two modules varied. Right panel: both intra-module and inter-module connectivities varied. Each plot shows mean RMSE of 100 runs, where error bars indicate sample standard deviations in each side. Four methods are shown with different colors and marker symbols. (b) Identical as above but for second component.

We also made a more quantitative comparison among the methods (Simulation II). Fig 4 shows the distributions of the estimation error at three different sample sizes: *N* = 100, 1000, and 10000. The two subfigures, (a) and (b), correspond to the first and second components in two different conditions: when only inter-module connectivity varied (left panel) or when both intra- and inter-module connectivity varied (right panel).

As seen in Fig 4(a) (left), both OCF and MCF decreased the error more quickly (in the sample size) than PCA. This clearly shows that these approaches may reduce the necessary sample size to reliably estimate large-scale eigenconnectivity patterns. However, Fig 4(a) (right) shows that OCF did not decrease the error even when the sample size became large when the intra-module connectivity additionally varied, which is consistent with the above illustrative example. In both conditions, MCF consistently exhibited the lowest errors. The error even decreased from that of the initial stepwise solution by Stepwise MCF (labeled “Init.” in the figure), at least when both intra- and inter-module connectivity varied. This demonstrates the advantage of the principled constrained PCA approach over the computationally simpler post-hoc matrix factorization approach. Qualitatively similar results were seen in the second component (Fig 4(b)), while the error slightly increased from that of the first component, especially at relatively small sample sizes.

### 3.2 Resting-state fcMRI data analysis

#### 3.2.1 Comparison with OCF

**Fig 5:**
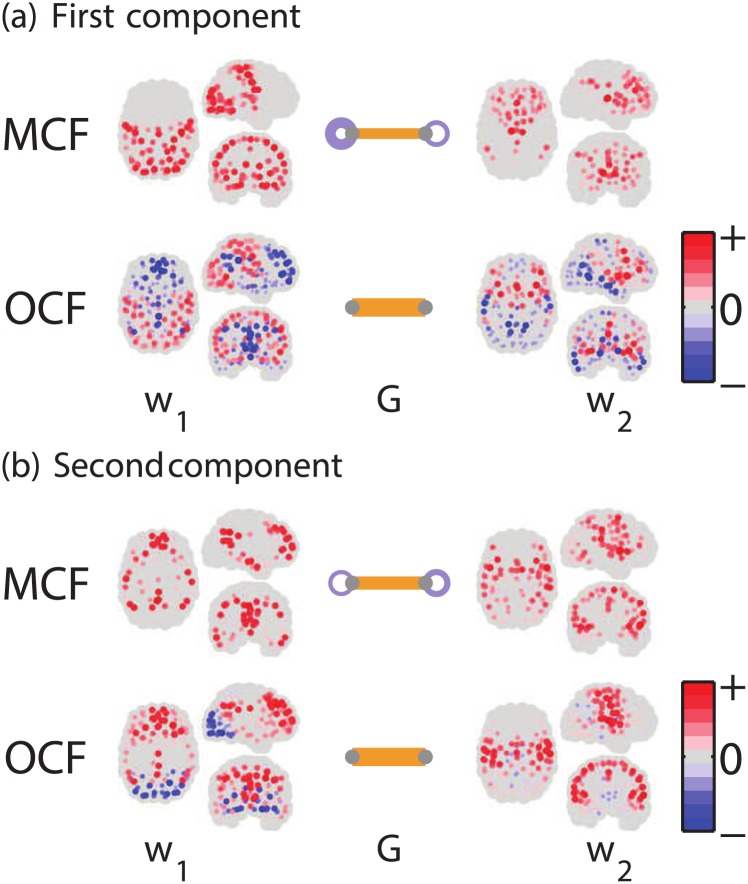
Resting-state fcMRI data. Spatial patterns of weight vector pairs, ***w***_1_ and ***w***_2_, as well as eigenconnectivity ***G*** between the two modules, obtained as (a) first and (b) second components of MCF and OCF. Dots display the magnitudes (indicated by color intensity and area and rescaled in each weight vector) and signs (red: positive, blue: negative) of weight values at 177 ROIs. The brain is transparently viewed from right lateral, dorsal, and posterior side. At center of each pair, eigenconnectivity ***G*** is represented as a two-node undirected graph. Magnitude $g_{kl}^{2}$ of each entry is indicated by width of edge (straight bars: off-diagonal entries, loops: diagonal entries); signs are indicated by two colors (orange: positive, purple: negative).

First we show the results by MCF of *K* = 2 in comparison to OCF. Fig 5(a) and 5(b), compare the first and second components, each of which were both obtained by MCF and OCF. These components explain the inter-individual variabilities in the connectivity matrices. The order of the two weight vectors in each method is actually arbitrary; we reordered the two OCF’s weight vectors to maximize their absolute inner products with the corresponding MCF’s weight vectors. The signs in the OCF’s weight vectors were also appropriately flipped so that the patterns are comparable between the two methods.

In both components, each spatial pattern obtained by MCF apparently consists of a few spatially localized submodules.

In the first component (Fig 5(a)) of MCF, ***w***_1_ forms an interesting network of primary sensory areas, mainly of visual and sensorimotor modalities, with additional participation from the auditory areas. This network is characterized by a large variability of intra-module connectivity, reflected in a large diagonal entry in ***G*** (see below).

The ***w***_2_ network in the first MCF component is characterized by large weights on the subcortical areas (thalamus and basal ganglia), while other areas in the frontal cortex, the posterior cingulate cortex (PCC), and the angular gyri (AG) also participated. We do not have a clear interpretation for this network, which seems to combine elements of the executive control and the default-mode network (DMN) with subcortical structures.

In the second MCF component (Fig 5(b)), ***w***_1_ strikingly resembles the DMN, including ACC, AG, and the medial prefrontal cortex as well as parts of the medial temporal lobe. ***w***_2_ seems to be a combination of the task-positive network (TPN) [28] and the salience network [29].

The spatial patterns of the positive weights (red) in OCF resemble those of MCF. However, the OCF patterns combine negative weights (blue) and positive weights, which may complicate interpretation. The negative weights of ***w***_1_ in the first component again resemble the DMN, while the positive weights resemble the general sensory areas network in MCF. In ***w***_2_, the positive parts appear to form the salience network, while the negative parts may be a purely visual network. Thus, in this result, each OCF pattern seems to simultaneously represent two distinct functional (sub)networks in the positive and negative parts of the weight vector. Readers might wonder why the OCF results look different from the original results [18], obtained for a subset (103 subjects) of our full dataset. However, the difference is actually not qualitatively large after appropriately flipping the signs (specifically those of ***w***_1_ in Fig 5(a) and ***w***_2_ in Fig 5(b) of our OCF results. Note that the signs of the weight vectors are arbitrary in OCF due to the inherent indeterminacy.

The module-level eigenconnectivity, ***G***, illustrated as a two-node undirected graph at the center, further indicates that the variability within some modules was not negligible. The different signs between the intra-and inter-module eigenconnectivity in both the first and second MCF components mean that these component explains anti-correlated changes between the two types of connectivities. As mentioned above, the first MCF component (Fig 5(a)) exhibited almost the same order of intra-module variability $g_{11}^{2}$ (in the module represented by ***w***_1_) as that of the variability of inter-module connectivity $g_{12}^{2}$. This is easy to understand since the network in the ***w***_1_ groups together sensory areas of different modalities, and the connectivities among the sensory modalities are probably quite variable. In contrast, as illustrated by the simulation study in Section 2.4.1, we must be careful about the interpretation of the OCF results when the intra-module network variability is not negligible.

#### 3.2.2 Further demonstration with more than two modules

Our MCF can be generalized with more than two modules (Section 2.3.5). Here, we see that using a higher *K* may increase the neurophysiological interest and ease interpretation.

**Fig 6:**
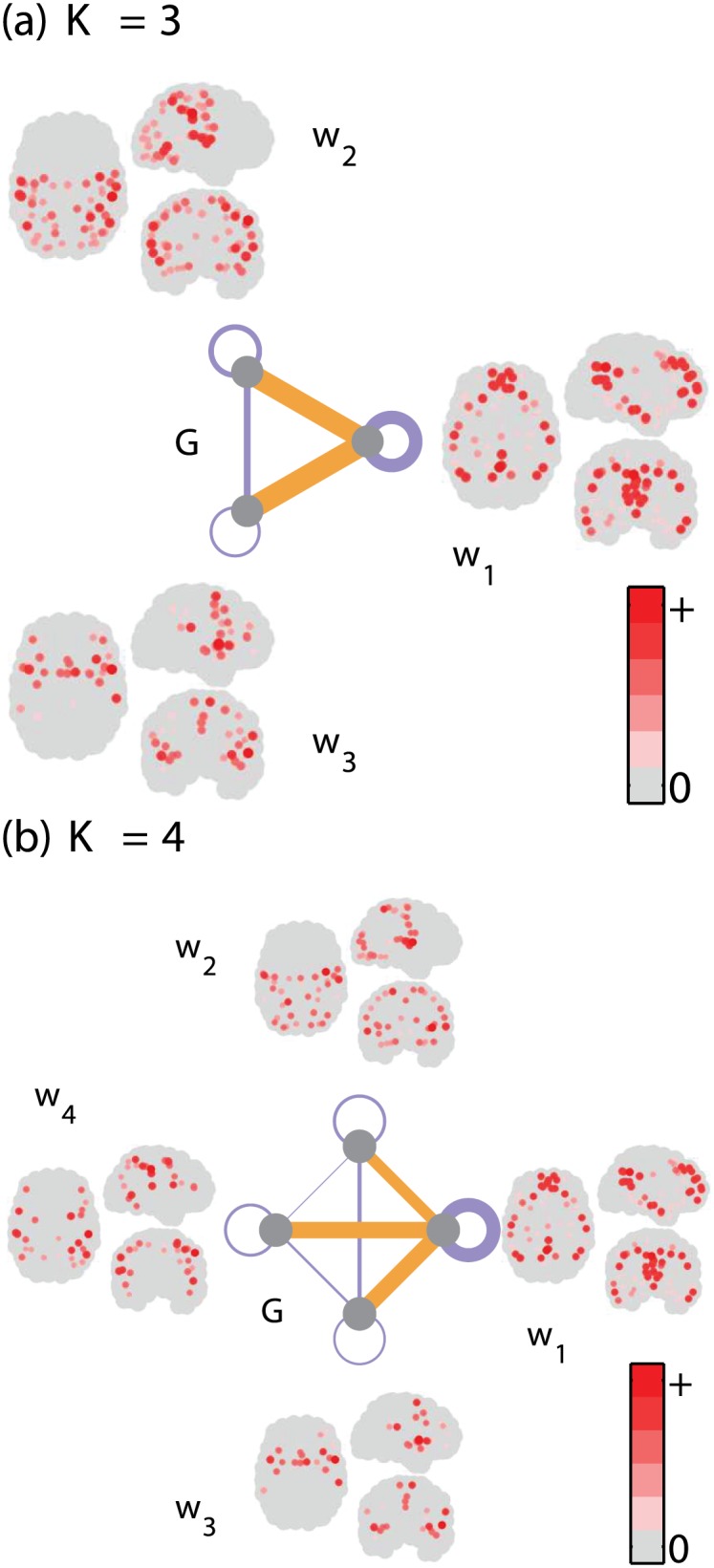
Resting-state fcMRI data. Spatial patterns of weight vectors ***w***_*k*_ as well as module-level eigenconnectivity ***G***, obtained as first components of MCF with number of modules (a) *K* = 3 and (b) *K* = 4. See caption of Fig 5 for visualization details.

We first show the weight and module-level eigenconnectivity patterns for *K* = 3 (Fig 6(a)). First, ***w***_1_ seems to represent the DMN, possibly with some additional temporal contribution. Second, ***w***_2_ seems to be a general sensory area network, similar to ***w***_1_ in Fig 5(a), with slightly less emphasis on the visual and anterior sensorimotor areas. Finally, ***w***_3_ resembles the salience network [29] with a particular contribution from the insula.

Thus, the three networks seem to represent clearly defined networks, two of which are well-known (salience and DMN) and one of which is new but reasonable (sensory areas). But what is even more interesting is that this particular combination of three networks appears very logical in the following sense; the sensory area network is related to outward-directed information processing, and the DMN is related to inward-directed information processing, while the salience network switches between the two, in particular, detecting when new significant sensory information starts arriving. In fact, the connectivities of the salience network to the DMN and the sensory network have opposite signs.

When the number of modules (networks) is increased to *K* = 4 (Fig 6(b)), the three networks found for *K* = 3 are mainly intact, but we see a new network in ***w***_4_ that seems to be a very clear-cut TPN with contributions around but not including the central sulcus, similar to [28]. It is also interesting that the DMN’s intra-module variability is rather large compared to all the others. This is presumably due to its different parts (e.g., anterior vs. posterior parts) that perform different functions and thus with variable connectivities [5, 30]. Additional results by Stepwise MCF and another stepwise analysis using *k*-means clustering is given in S2 Appendix; a further comparison with an existing nonnegative tensor factorization method is found in S3 Appendix.

In this analysis, the running time of MCF to extract the 1st component (*K* = 4) was 1.55 ± 0.01s (mean±SD, 20 runs) on a Linux computer with 18 cores, 2.30GHz CPU and 128GB RAM, while performing only Stepwise MCF took 1.27 ± 0.01s.

## 4 Discussion

The brain’s functional architecture varies across subjects and/or over time, and its statistical characterization is crucial to enhance our neuroscientific understanding of, e.g., individual difference in cognitive functions or temporal changes in mental states, as well as for potential clinical applications. Although PCA is a fundamental method to characterize the variability of functional connectivity matrices [8], it has a severe limitation in visualization and interpretation of complicated eigenconnectivity patterns.

To overcome this issue, we extended the idea of OCF [18] and developed MCF as a novel module-constrained PCA method which explicitly takes the brain’s functional modularity into account. As demonstrated in our resting-state fcMRI analysis (Section 3.2), MCF’s factorized representation of eigenconnectivity matrix can be intuitively visualized using spatial patterns of functional modules (networks) as well as a graph that summarizes the eigenconnectivity at the module level. Such a ease of visualization is a great advantage of MCF over PCA. The simulation studies (Section 3.1) also showed that MCF even needs less sample sizes for estimation and our principled constrained PCA approach is advantageous over a post-hoc analysis of PCA eigenconnectivity matrices.

Our MCF improved OCF so that it is readily applicable even when the variability in intra-module connectivity is not negligible. The simulations and fcMRI data analysis clearly showed the advantage of explicitly taking the intra-module network variability into account as well as improved interpretability by additional nonnegativity constraints. We also demonstrated that generalization to more than two modules can further increase the neurophysiological interest and ease interpretation. From the above results, we conclude that our MCF improves both interpretability and applicability over PCA and OCF and is a promising alternative to these methods to explore the random variability of functional brain connectivity.

Applications of MCF to actual neuroscience or clinical studies will be a promising next step of the present study. For example, the method will be useful to identify modules and their eigenconnectivity that may have different contributions to functional connectivity in different populations, such as patients and controls. The improved interpretability and applicability of MCF then will be beneficial to draw meaningful conclusions more effectively. In particular, the compact module-level representation may ease the visualization of results, avoiding additional rather complicated post-processing steps about node ordering or edge pruning. This may greatly improve the overall efficiency of the analysis and may even reduce the arbitrariness in the post-processing.

In practice, many techniques other than PCA, such as *k*-means clustering [7, 9] and independent component analysis (ICA) [12], can also be used to summarize connectivity matrices. One may also develop module-constrained versions of these methods by incorporating the same factorized matrix representation ***B*** = ***WGW***^⊤^ as used by MCF, where ***B*** would be a cluster centroid of *k*-means or a basis element of ICA; we have already done a stepwise analysis corresponding to such an approach with a promising result (S2 Appendix). To derive specific algorithms, one can use a similar lower-bounding technique (as Eq (10)) to derive an alternating optimization involving the subproblem Eq (11). We leave the investigation on this topic for future research.

The method presented here is actually very general and may have a wide range of applicability beyond such applications to functional brain connectivity data. In fact, since MCF does not rely on the positive definiteness of observed connectivity matrices, it is applicable to any random network data and is not limited to covariance (correlation) matrices as is typical in functional connectivity analysis. In brain imaging applications, such data include anatomical connectivity measured by diffusion tensor imaging (DTI). On the other hand, explicit use of the geometric structure of positive semidefinite matrices might further improve our method when restricting the data type as covariance or correlation matrices (e.g., [31]).

From a broader perspective, statistical modeling and the analysis of time-varying (nonstationary) networks are a topic of great interest in machine learning and network science. Many authors have developed methods, e.g., based on nonstationary graphical Markov modeling with temporal smoothness [32–36], or state-space modeling combined with so-called stochastic blockmodels or related probabilistic models [37–40], whose applications to brain imaging data can also be found (e.g., [41, 42]). In contrast to such model-based methods, PCA-based eigenconnectivity analysis like MCF puts more emphasis on extracting the relevant aspects of data in a condensed manner rather than fully modeling and predicting the network (connectivity) changes. It can even analyze purely inter-subject variability as demonstrated, since it does not rely on any temporal structure (like smoothness) in the data.

In the present study, we did not mention some important conceptual aspects of OCF at all. For example, a generative model interpretation of OCF for covariance or correlation matrices has also been presented [18] in which the original data vectors (from which the sample covariances or correlations are computed) are generated by a linear factor-analytic model with orthogonal loadings. In addition, OCF has close connections to machine learning techniques other than nonnegative tensor factorization, such as common spatial patterns and blind source separation methods, as extensively discussed [18]. Since MCF shares a fundamental framework with OCF, similar interpretations and connections are expected to be valid for MCF. Detailed investigation is left for future study.

A Matlab software implementing our method will be available at the first author’s website (http://www.cns.atr.jp/~hirayama).

## Supporting Information

S1 Appendix Proofs and derivations. (PDF)

S2 Appendix Additional results by stepwise analysis. (PDF)

S3 Appendix Relation to Nonnegative Tensor Factorization. (PDF)
