# Supplementary material for: Characterizing Variability of Modular Brain Connectivity with Constrained Principal Component Analysis
Source: PLoS One. 2016 Dec 21;11(12):e0168180. doi: 10.1371/journal.pone.0168180 (PMC5176286; doi:10.1371/journal.pone.0168180)
Supplement: S1 Appendix — (PDF) [file pone.0168180.s001.pdf]

S1 Appendix
Proofs and derivations

A Derivation of Stepwise MCF algorithm

Our Stepwise MCF performs a novel post-hoc analysis of the eigenconnectivity matrix $\boldsymbol{B}_{\mathrm{PCA}}$ obtained by unconstrained PCA. Specifically, we seek an approximate factorization $\boldsymbol{B}_{\mathrm{PCA}} \approx \boldsymbol{W}\boldsymbol{G}\boldsymbol{W}^\top$ where $\boldsymbol{W}$ and $\boldsymbol{G}$ satisfy their respective constraints introduced in Section 2.3.2. In the following, we derive the algorithm using a spectral relaxation technique similar to the one developed in the literature of (multiclass) spectral clustering [1].

Assume for a moment that weight matrix $\boldsymbol{W}$ can be any matrix satisfying relaxed constraint $\boldsymbol{W}^\top\boldsymbol{W} = \boldsymbol{I}$. Then any $\boldsymbol{W}$ and $\boldsymbol{G}$, such that $\boldsymbol{W}\boldsymbol{G}\boldsymbol{W}^\top = \boldsymbol{U}\boldsymbol{Q}\boldsymbol{U}^\top$, achieves the minimum approximation error $\|\boldsymbol{B}_{\mathrm{PCA}} - \boldsymbol{W}\boldsymbol{G}\boldsymbol{W}^\top\|^2$, where diagonal matrix $\boldsymbol{Q}$ contains the two largest-magnitude eigenvalues of $\boldsymbol{B}_{\mathrm{PCA}}$ and $D \times 2$ matrix $\boldsymbol{U}$ has corresponding eigenvectors in its columns. The relaxed solutions are thus given by $\boldsymbol{W} = \boldsymbol{U}\boldsymbol{V}^\top$ with arbitrary 2×2 orthogonal matrix $\boldsymbol{V}$.

Although these relaxed solutions do not necessarily contain any solution under the original constraints, we might still expect to obtain a reasonable partitioning into modules (with nonnegative weights) by finding the $\boldsymbol{W}$ that is closest to the set of relaxed solutions among those satisfying original constraints. For convenience, we define $\Omega_+ := \Omega \cap \mathbb{R}_+^{D\times 2}$. Then the $\boldsymbol{W} \in \Omega_+$ closest to any relaxed solution may be found by solving the following minimization problem:

$$\begin{aligned} \min_{\boldsymbol{W},\boldsymbol{V}} \quad & \|\boldsymbol{W} - \boldsymbol{U}\boldsymbol{V}^\top\|^2, \\ \text{subject to} \quad & \boldsymbol{W} \in \Omega_+, \quad \boldsymbol{V}^\top\boldsymbol{V} = \boldsymbol{I}. \end{aligned} \tag{1}$$

After solving this, we simply normalize every column of $\boldsymbol{W}$ to satisfy all of the original constraints, i.e., $\boldsymbol{W} \in \Omega_+$ and $\forall k\ \|\boldsymbol{w}_k\| = 1$. Note that the solution is only an approximation to the exact orthogonal projection to the set of $\boldsymbol{W}$s satisfying both of these two constraints (see also Section 2.3.4), while it empirically works quite well in our experiments. Finally, for the given $\boldsymbol{W}$, we set $\boldsymbol{G} = \boldsymbol{W}^\top\boldsymbol{B}_{\mathrm{PCA}}\boldsymbol{W}$ to obtain the best approximation $\boldsymbol{B}_{\mathrm{PCA}} \approx \boldsymbol{W}\boldsymbol{G}\boldsymbol{W}^\top$.

The problem (1) is the same as a previously considered one [1], except for the explicit nonnegativity on $\boldsymbol{W}$. Following [1], we solve (1) using an alternating minimization between $\boldsymbol{W}$ and $\boldsymbol{V}$. The solution for $\boldsymbol{W}$ is obtained with Proposition 1 below; the other problem for $\boldsymbol{V}$ is the well-known orthogonal Procrustes problem [2] for which the solution is given by $\boldsymbol{V} = \boldsymbol{R}\boldsymbol{L}^\top$, where $\boldsymbol{L}\boldsymbol{\Sigma}\boldsymbol{R}^\top$ is the singular value decomposition of $\boldsymbol{U}^\top\boldsymbol{W}$.

Proposition 1. *Let* $\widehat{\boldsymbol{W}} = \mathcal{P}_{\Omega_+}(\boldsymbol{W}) := \operatorname{argmin}_{\boldsymbol{W}'\in\Omega_+} \|\boldsymbol{W}' - \boldsymbol{W}\|^2$ *be the orthogonal projection of* $\boldsymbol{W} = (w_{jk})$ *to set* Ω_+ *defined in* (8). *Then it holds that the* (j,k)*-element of* $\widehat{\boldsymbol{W}}$ *is given by*

$$\widehat{w}_{jk} = \begin{cases} (w_{jk})_+ & \text{if } k = k_j^* \\ 0 & \text{otherwise} \end{cases}, \tag{2}$$

where $k_j^* = \operatorname{argmax}_k w_{jk}$ *for every* j *and* $(w)_+ := \max\{w, 0\}$.

Proof. Let $\kappa : j \mapsto k$ indicate module k to which node j belongs. Then for any given κ, we have

$$\begin{aligned}\min_{\boldsymbol{W}'} \|\boldsymbol{W}' - \boldsymbol{W}\|^2 &= \sum_j \left\{ \sum_{k \neq \kappa(j)} w_{jk}^2 + \min_{w'_{j\kappa(j)} \geq 0} (w'_{j\kappa(j)} - w_{j\kappa(j)})^2 \right\} \\ &= \sum_j \left\{ \sum_{k \neq \kappa(j)} w_{jk}^2 + (-w_{j\kappa(j)})_+^2 \right\} \\ &= \|\boldsymbol{W}\|^2 - \sum_j (w_{j\kappa(j)})_+^2,\end{aligned}$$

where $w'_{j\kappa(j)} = (w_{j\kappa(j)})_+$ solves inner minimization. Further minimizing this with respect to κ, we obtain $\kappa(j) = \mathrm{argmax}_k \, w_{jk}$ for every j, which concludes the proof. $\square$

B Alternative interpretation of problem (9)

Although we have introduced the optimization problem (9) solely as a constrained form of the original PCA optimization problem (2), one may also view the same problem from another perspective. To see this, denote by $\boldsymbol{Y} := \boldsymbol{W}^\top \boldsymbol{X} \boldsymbol{W}$ (2×2 matrix) the module-level summary of observed connectivity matrix, where each entry, $y_{kl} = \boldsymbol{w}_k^\top \boldsymbol{X} \boldsymbol{w}_l$, summarizes the total (weighted) connectivity within module k (if $k = l$) or between module k and module l (if $k \neq l$). Then, the problem (9) with respect to $\boldsymbol{G}$ reduces to the following PCA optimization problem just like (2):

$$\max_{\boldsymbol{G}} \sum_{n=1}^{N} \left(\mathrm{tr}[\boldsymbol{G}^\top \widetilde{\boldsymbol{Y}}_n] \right)^2, \quad \text{subject to} \quad \|\boldsymbol{G}\| = 1,$$

where $\widetilde{\boldsymbol{Y}}_n := \boldsymbol{Y}_n - \bar{\boldsymbol{Y}}$ and $\bar{\boldsymbol{Y}}$ denotes the sample mean; every $\widetilde{\boldsymbol{Y}}_n$ actually depends on $\boldsymbol{W}$ which is simultaneously optimized for the same objective. Hence, the constrained PCA problem (9) of our MCF can also be interpreted as performing PCA eigenconnectivity analysis at the module level, simultaneously optimizing the partitioning and weights of the modules in a unified manner.

C Derivation of gradient (13)

The gradient is calculated as

$$\begin{aligned}\nabla_{\boldsymbol{W}} \|\boldsymbol{W}^\top \boldsymbol{C} \boldsymbol{W}\|^2 &= \left(\mathrm{tr} \left[(2\boldsymbol{W}^\top \boldsymbol{C} \boldsymbol{W})^\top \frac{\partial}{\partial w_{kl}} \boldsymbol{W}^\top \boldsymbol{C} \boldsymbol{W} \right] \right)_{kl} \\ &= \boldsymbol{C}\boldsymbol{W}(2\boldsymbol{W}^\top \boldsymbol{C} \boldsymbol{W})^\top + \boldsymbol{C}^\top \boldsymbol{W}(2\boldsymbol{W}^\top \boldsymbol{C} \boldsymbol{W}) \\ &= 2\boldsymbol{C}\boldsymbol{W}\boldsymbol{W}^\top \boldsymbol{C}^\top \boldsymbol{W} + 2\boldsymbol{C}^\top \boldsymbol{W}\boldsymbol{W}^\top \boldsymbol{C}\boldsymbol{W},\end{aligned}$$

where we used relation $\nabla_{\boldsymbol{W}} \mathrm{tr}[\boldsymbol{A}\boldsymbol{W}^\top \boldsymbol{C}\boldsymbol{W}] = \boldsymbol{C}\boldsymbol{W}\boldsymbol{A} + \boldsymbol{C}^\top \boldsymbol{W}\boldsymbol{A}^\top$ for any constant matrix $\boldsymbol{A}$. Then the symmetricity of $\boldsymbol{C}$ implies (13).

References

[1] Yu SX, Shi J. Multiclass spectral clustering. In: Proceedings of the Ninth IEEE International Conference on Computer Vision (ICCV2003); 2003. p. 313–319.

[2] Schönemann PH. A generalized solution of the orthogonal Procrustes problem. Psychometrika. 1966;31:1–10.
