# Supplementary material for: Characterizing Variability of Modular Brain Connectivity with Constrained Principal Component Analysis
Source: PLoS One. 2016 Dec 21;11(12):e0168180. doi: 10.1371/journal.pone.0168180 (PMC5176286; doi:10.1371/journal.pone.0168180)
Supplement: S2 Appendix — (PDF) [file pone.0168180.s002.pdf]

S2 Appendix
Additional results by stepwise analysis

Methods

We additionally performed a stepwise analysis on the resting-state fcMRI data (Section 3.2.2) using Stepwise MCF (Algorithm 1). For reference, we also performed a stepwise analysis combined with k-means clustering, by performing it at the line 1 of Algorithm 1 instead of PCA. We set the number of clusters at two for a simple demonstration, and post-processed the two cluster centroids individually by Algorithm 1. In all the analysis, the number of modules was set to $K = 4$ as in Fig. 6(b) by MCF.

Results

The figure below visualizes the spatial patterns of modules and their eigenconnectivity matrices obtained by the stepwise analysis methods.

In (a), Stepwise MCF found the two modules, DMN (at the right) and TPN (left), with almost the same spatial patterns as those in Fig. 6(b). The other two modules (top and bottom) also have some similarity with the corresponding ones, but both spatial patterns are noisier than those in Fig. 6(b). The result is reasonable since Stepwise MCF may suffer from a larger variance of unconstrained PCA unlike MCF. The module-level eigenconnectivity $\boldsymbol{G}$ also differs from the one in Fig. 6(b) accordingly.

In (b), the additional stepwise analysis using k-means clustering also produced characteristic modules and their intra- and inter-connections. It is readily seen that both centroids contain a module that resembles DMN (at the right of each graph), implying that DMN is a very stable structure. A module resembling TPN is also found in the first centroid. Although full investigations of the modules and their connections are out of the scope of the present study, the result suggests that neurophysiologically meaningful modules may emerge even when the idea of MCF is combined with methods other than PCA to summarize connectivity matrices.

(a) PCA (Stepwise MCF)

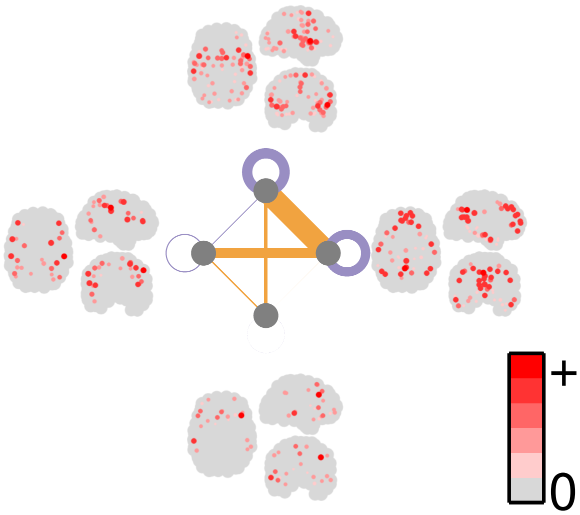

(b) k-means ($k = 2$)

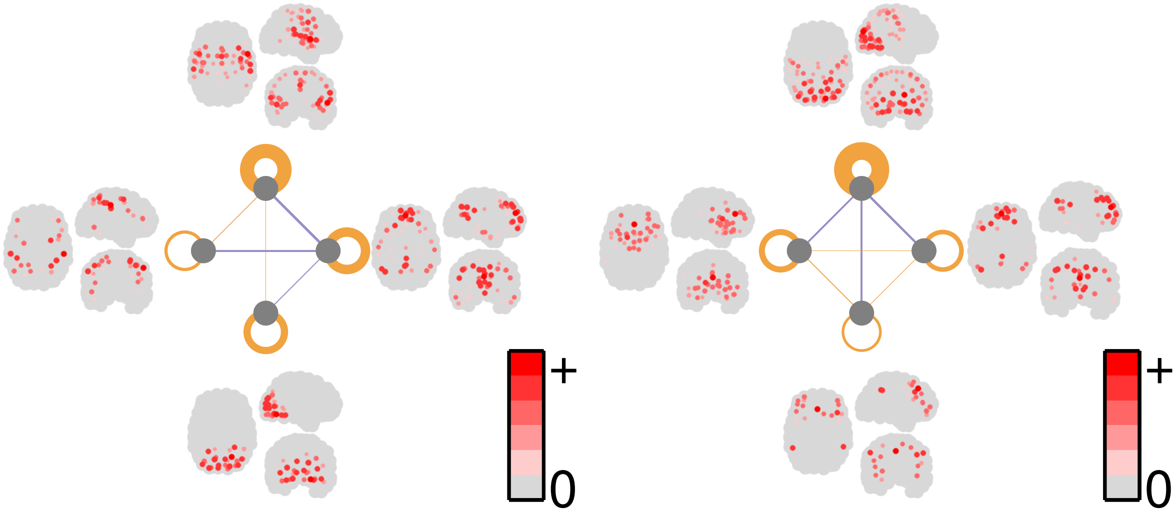

Figure. Resting-state fcMRI data: Spatial patterns of weight vectors $\boldsymbol{w}_k$ as well as module-level eigenconnectivity $\boldsymbol{G}$ of obtained by stepwise analysis. See the caption of Fig. 5 for visualization details. (a) The first component obtained by the PCA-based Stepwise MCF (Algorithm 1). (b) The two centroids obtained by replacing PCA with k-means clustering (two clusters). The global signs of $\boldsymbol{G}$ were not adjusted here, as they are not arbitrary for clustering unlike PCA.
