# Supplementary material for: Characterizing Variability of Modular Brain Connectivity with Constrained Principal Component Analysis
Source: PLoS One. 2016 Dec 21;11(12):e0168180. doi: 10.1371/journal.pone.0168180 (PMC5176286; doi:10.1371/journal.pone.0168180)
Supplement: S3 Appendix — (PDF) [file pone.0168180.s003.pdf]

S3 Appendix
Relation to Nonnegative Tensor Factorization

A MCF and Nonnegative Tucker decomposition (NTD)

We developed our MCF method as a new constrained PCA for connectivity matrix data. However, it turns out that MCF also has a close relationship to nonnegative Tucker decomposition (NTD) [1, 2], a specific type of nonnegative tensor factorization (see, e.g., [3]), due to the additional nonnegativity constraint as well as the generalization to more than two modules; NTD is a generalization of a more conventional parallel factor analysis (PARAFAC) type of method that has recently been applied to the brain's functional network analysis [4].

To introduce the basic idea of NTD, let $\boldsymbol{\mathcal{X}} \in \mathbb{R}^{D\times D\times N}$ be a three-dimensional array (i.e., third-order tensor) that concatenates connectivity matrices $\boldsymbol{X}_n$ along the third dimension. Then NTD seeks an approximate decomposition of tensor $\boldsymbol{\mathcal{X}}$ such that

$$\boldsymbol{\mathcal{X}} \approx \sum_{k=1}^{K}\sum_{l=1}^{L}\sum_{m=1}^{M} g_{klm}\boldsymbol{w}_k \otimes \boldsymbol{w}'_l \otimes \boldsymbol{s}_m, \tag{1}$$

where $\boldsymbol{w}_k$ and $\boldsymbol{w}'_l$ are D-dimensional nonnegative vectors, $\boldsymbol{s}_m$ is N-dimensional nonnegative vector and $\otimes$ denotes an outer product of two vectors. Standard NTD sets coefficients g_{klm} as nonnegative as well, while a variant with real coefficients g_{klm} is usually called semi-nonnegative Tucker decomposition (sNTD). Algorithms for NTD and sNTD have been extensively studied in the literature (e.g., [3, 5]).

Now consider the case of $K = L$. Then it follows that sNTD approximates each connectivity matrix $\boldsymbol{X}_n$ as

$$\begin{aligned} \text{sNTD:} \quad \boldsymbol{X}_n &\approx \sum_{m=1}^{M} s_{mn}\boldsymbol{W}\boldsymbol{G}_m(\boldsymbol{W}')^\top, \\ s_{mn} &\geq 0, \quad \boldsymbol{W}, \boldsymbol{W}' \in \mathbb{R}_+^{D\times K}, \end{aligned} \tag{2}$$

where s_{mn} denotes the n-th entry of $\boldsymbol{s}_m$, matrices $\boldsymbol{W}$ and $\boldsymbol{W}'$ consist of column vectors $\boldsymbol{w}_k$ and $\boldsymbol{w}'_l$, and matrix $\boldsymbol{G}_m$ consists of entries g_{klm}. Due to the scaling ambiguity, we may set every column in $\boldsymbol{W}$ and $\boldsymbol{W}'$ to have unit norm and matrix $\boldsymbol{G}$ to have unit Frobenius norm. Equation (2) actually exhibits many similarities with the decomposition obtained by MCF after M-th deflation step, i.e.,

$$\begin{aligned} \text{MCF:} \quad \boldsymbol{X}_n &\approx \bar{\boldsymbol{X}} + \sum_{m=1}^{M} s_{mn}\boldsymbol{W}_m\boldsymbol{G}_m\boldsymbol{W}_m^\top, \\ s_{mn} &\in \mathbb{R}, \quad \boldsymbol{W}_m \in \Omega_+, \end{aligned} \tag{3}$$

where the subscript m indicates corresponding deflation steps and definition of set Ω_+ is generalized to $D \times K$ matrices.

Next, we discuss their differences in more detail. The typical sNTD decomposition (2) does not assume $\boldsymbol{W} = \boldsymbol{W}'$, while MCF does. However, this difference is not essential because the symmetry of connectivity matrices $\boldsymbol{X}_n$ again implies that one may reasonably set $\boldsymbol{W} = \boldsymbol{W}'$ and also set $\boldsymbol{G}$ as symmetric in (2) either by additional constraints or as a consequence of optimization. Other more essential differences between the decompositions by sNTD (2) and MCF (3) are the following:

1. *Parts-based representation vs. variability around mean.* sNTD directly decomposes connectivity matrices while MCF decomposes their deviations from mean. In other words, sNTD primarily seeks parts-based

representations of data rather than analyzing underlying factors of variability, just like what nonnegative matrix factorization (NMF) [6, 7, 3] does as compared to PCA. Note that the nonnegative components s_{mn} in sNTD naturally quantifies each part's contribution while nonnegativity is hardly justified when representing the variability around mean; subtraction of sample mean $\bar{\boldsymbol{X}}$ is usually not performed in the literature of nonnegative tensor factorization since it tends to destroy the nonnegativity of the data.

2. *Disjointness among modules.* In addition, MCF explicitly introduces disjointness among modules as well as nonnegativity, so that each entry of $\boldsymbol{G}_m$ clearly corresponds to either intra-module or inter-module variability. Although disjointness (i.e., orthogonality) of nonnegative weights has been studied extensively for NMF (e.g., [8, 3]), it does not seem to be very common for nonnegative tensor factorization (but see, e.g., [9, 10]). In particular, we are not aware of any studies using semi-nonnegative Tucker-type decomposition with additional disjointness constraints.

3. *Common modules across components.* Another difference is whether module weights $\boldsymbol{W}$ are common across components or not. MCF allows the modules to be different across components, which is more flexible and even enables to use the computationally simple deflation scheme. On the other hand, sNTD assumes that every component has the same collection of modules. Although being less flexible, such an assumption possibly improves the identification of relevant modules by reducing the number of free parameters.

Among these points, the first point is arguably the most important. The two types of decompositions by MCF and sNTD are essentially very different in their underlying concepts and hence, sNTD is not directly applicable to eigenconnectivity analysis. We do not claim that either of the two approaches is better than the other. They actually focus on rather different aspects of data and can work complementary.

B Additional result by sNTD

For comparison with the multi-module generalization of MCF ($K = 4$), we applied sNTD to the same fcMRI dataset (Section 2.5.1) to extract a single component (i.e., $M = 1$) with $K = L = 4$. We used a Matlab implementation of an efficient hierarchical alternating least squares (HALS) algorithm [5] [1]. To avoid local optima, we ran the algorithm 100 times from different initial conditions to converge and selected a result that achieved the smallest reconstruction error. In the 100 runs, the solution always approximately satisfied $\boldsymbol{W} = \boldsymbol{W}'$ after appropriate reordering of the columns in $\boldsymbol{W}'$ and rescaling all the columns in $\boldsymbol{W}$ and $\boldsymbol{W}'$ to have unit norm; inner products of the corresponding unit vectors $\boldsymbol{w}_k$ and $\boldsymbol{w}'_k$ were 0.99 on average. The solution $\boldsymbol{G}$ after corresponding permutation and scaling were also always approximately symmetric; the relative error by $\|\boldsymbol{G} - \boldsymbol{G}^\top\|^2/\|\boldsymbol{G}\|^2$ was 9.40×10^{-4} on average. For visualizing results, we used only $\boldsymbol{W}$ by discarding $\boldsymbol{W}'$ and explicitly symmetrized $\boldsymbol{G}$ by taking average with its transpose.

As is normally done (e.g., [2]), we performed sNTD on non-centered connectivity matrices $\boldsymbol{X}_n$, for which the decomposition with nonnegative components (2) is well-justified. For completeness, we also intentionally performed sNTD on centered connectivity matrices $\widetilde{\boldsymbol{X}}_n$, which we refer to as sNTD-c below. Note that such a usage is not common in the literature.

The result is shown in the figure given below. In Fig. A(a), $\boldsymbol{w}_1$ and $\boldsymbol{w}_3$ clearly resemble the DMN and the salience network which were also found by MCF, while $\boldsymbol{w}_2$ and $\boldsymbol{w}_4$ are sensorimotor and visual areas networks which might jointly correspond to the single network $\boldsymbol{w}_2$ in Fig. 6(b) but with a more spatial extent. Interestingly, sNTD produced no modules that resembled the TPN like $\boldsymbol{w}_4$ in Fig. 6(b). It is also seen that $\boldsymbol{G}$ has dominant diagonal entries as indicated by the thicker self-loops, and thus did not find interesting any inter-module relationship.

The modules were surprisingly unchanged even when the sample mean was subtracted in sNTD-c (Fig. A(b)). sNTD-c seems to find partial eigenconnectivity patterns of MCF like those within and between the DMN $\boldsymbol{w}_1$ and the salience network $\boldsymbol{w}_3$, while one must be careful about the interpretation of $\boldsymbol{G}$ as sNTD-c does not actually maximize the sample variability (variance) of connectivity matrices.

[1] Available at http://www.bsp.brain.riken.jp/˜zhougx/tensor.html

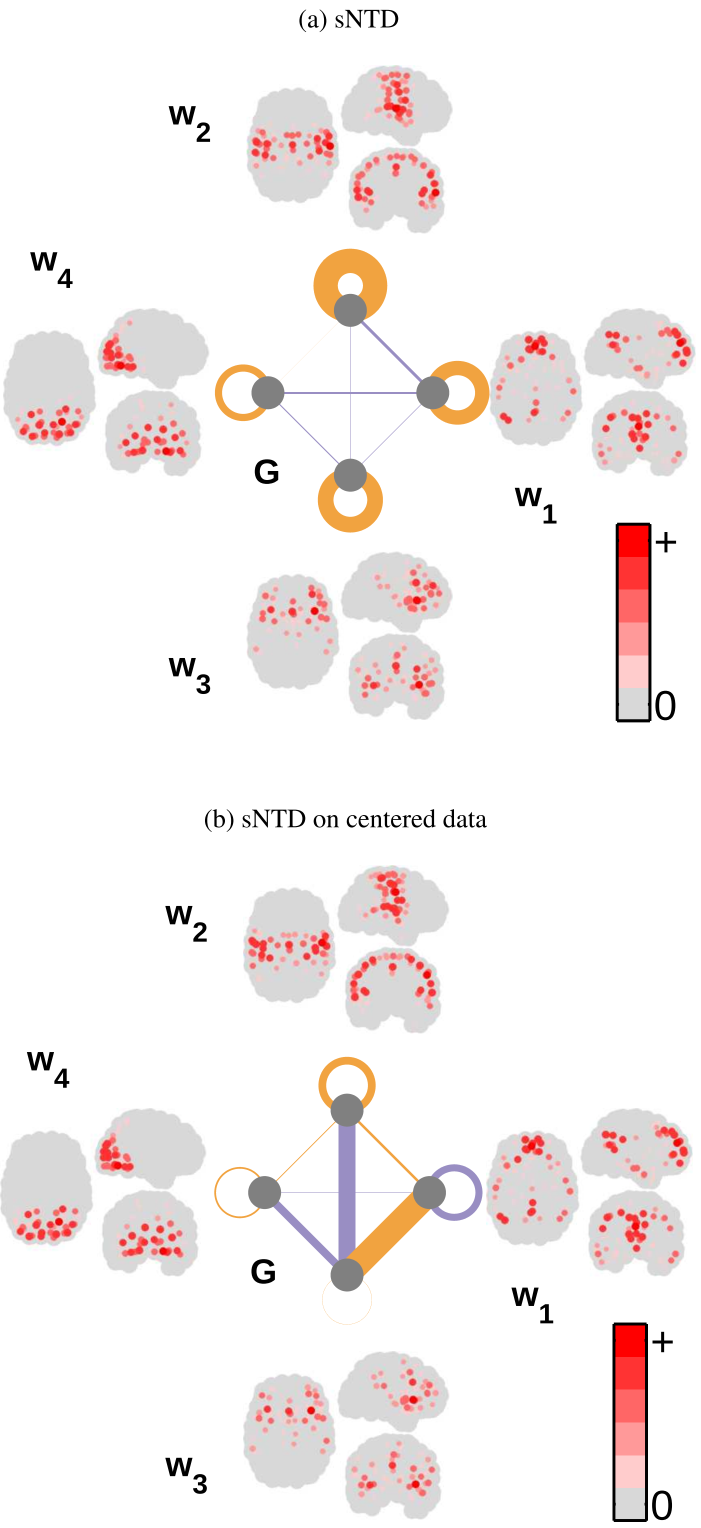

Fig A. Resting-state fcMRI data: spatial patterns of weight vectors $\boldsymbol{w}_k$ as well as module-level eigenconnectivity $\boldsymbol{G}$, obtained by (a) semi-nonnegative Tucker decomposition (sNTD) and (b) sNTD performed on centered data. Note that the latter usage is not common in the literature. See caption of Fig. 5 for visualization details.

References

[1] Kim YD, Choi S. Nonnegative Tucker Decomposition. In: IEEE Conference on Computer Vision and Pattern Recognition (CVPR '07); 2007. p. 1–8.

[2] Gauvin L, Panisson A, Cattuto C. Detecting the Community Structure and Activity Patterns of Temporal Networks: A Non-Negative Tensor Factorization Approach. PLoS ONE 9(1). 2014;9(1):e86028.

[3] Cichocki A, Zdunek R, Amari AHPS. Nonnegative Matrix and Tensor Factorizations: Applications to Exploratory Multi-way Data Analysis and Blind Source Separation. Wiley Publishing; 2009.

[4] Ponce-Alvarez A, Deco G, Hagmann P, Romani GL, Mantini D, Corbetta M. Resting-State Temporal Synchronization Networks Emerge from Connectivity Topology and Heterogeneity. PLoS Computional Biology. 2015;11(2):e1004100.

[5] Zhou G, Cichocki A, Shengli X. Fast Nonnegative Matrix/Tensor Factorization Based on Low-Rank Approximation. IEEE Transactions on Signal Processing. 2012;60(6):2928–2940.

[6] Paatero P, Tapper U. Positive matrix factorization: A non-negative factor model with optimal utilization of error estimates of data values. Environmetrics. 1994;5:111–126.

[7] Lee DD, Seung HS. Learning the parts of objects by non-negative matrix factorization. Nature. 1999;401(6755):788–791.

[8] Ding C, Li T, Peng W, Park H. Orthogonal Nonnegative Matrix Tri-Factorizations for Clustering. In: Proceedings of the 12th ACM SIGKDD international conference on Knowledge discovery and data mining (KDD2006); 2006. p. 126–135.

[9] Zdunek R. Uni-orthogonal Nonnegative Tucker Decomposition for Supervised Image Classification. In: Image Analysis and Processing – ICIAP 2011. vol. 6978 of Lecture Notes in Computer Science; 2011. p. 88–97.

[10] Zhang ZY, Li T, Ding C. Non-negative Tri-factor tensor decomposition with applications. Knowledge and Information Systems. 2013;34(2):243–265.
